# Catalyst-TiO(OH)_2_ could drastically reduce the energy consumption of CO_2_ capture

**DOI:** 10.1038/s41467-018-05145-0

**Published:** 2018-07-10

**Authors:** Qinghua Lai, Sam Toan, Mohammed A. Assiri, Huaigang Cheng, Armistead G. Russell, Hertanto Adidharma, Maciej Radosz, Maohong Fan

**Affiliations:** 1 College of Engineering and Applied Sciences, University of Wyoming, Laramie, WY 82071 USA; 2 College of Arts and Sciences, University of Wyoming, Laramie, WY 82071 USA; 3 School of Civil and Environmental Engineering, Georgia Institute of Technology, Atlanta, GA 30332 USA; 4 School of Energy Resources, University of Wyoming, Laramie, WY 82071 USA

## Abstract

Implementing Paris Climate Accord is inhibited by the high energy consumption of the state-of-the-art CO_2_ capture technologies due to the notoriously slow kinetics in CO_2_ desorption step of CO_2_ capture. To address the challenge, here we report that nanostructured TiO(OH)_2_ as a catalyst is capable of drastically increasing the rates of CO_2_ desorption from spent monoethanolamine (MEA) by over 4500%. This discovery makes CO_2_ capture successful at much lower temperatures, which not only dramatically reduces energy consumption but also amine losses and prevents emission of carcinogenic amine-decomposition byproducts. The catalytic effect of TiO(OH)_2_ is observed with Raman characterization. The stabilities of the catalyst and MEA are confirmed with 50 cyclic CO_2_ sorption and sorption. A possible mechanism is proposed for the TiO(OH)_2_-catalyzed CO_2_ capture. TiO(OH)_2_ could be a key to the future success of Paris Climat e Accord.

## Abstract (web summary)

The notoriously slow kinetics in CO_2_ desorption hinders the development of efficient CO_2_ capture technologies. Here, the authors discover that nanostructured TiO(OH)_2_ as a catalyst is capable of dramatically increasing the rates of CO_2_ desorption from spent monoethanolamine.

## Introduction

Carbon-based fuels have sustained the world for many centuries. However, their uses have resulted in large amounts of CO_2_ emission, a likely cause of the increasingly noticeable climate changes^1–4^. Thus, CO_2_ emission control is imperative by the Paris Climate Accord^5,6^. Chemisorption is the most selective and thus promising technology for capturing CO_2_ emitted from fossil flue gas due to its simplicity and unique flexibility in dealing with wide concentrations and volumes of CO_2_-containing gases^7–9^. However, its shortcomings are also obvious, including slow sorption and desorption rates. The desorption kinetics is much more important because the CO_2_ desorption step typically is much slower and more energy-intensive than the sorption step in the overall CO_2_ capture processes. The CO_2_ desorption step in conventional CO_2_ capture technologies has to be operated at higher temperatures for achieving higher reaction kinetics, typically above 100 °C, which makes this step very expensive, primarily due to the high specific and latent heat capacities of water^10^.

How can the absorbed CO_2_ be desorbed quickly at temperatures lower than 100 °C—the critical temperature for lowering energy consumption? If the use of catalyst can drastically enhance desorption rates, and thus allow for reducing the CO_2_ desorption temperatures, such low desorption temperatures would allow not only for reducing the energy consumption but also utilizing the existing flue-gas waste heat or solar-heated-water, which could completely eliminate the extra energy sources otherwise needed for conventional above 100 °C-desorption based CO_2_ capture technologies.

A few studies have shown that inorganic materials could be used as catalysts for CO_2_ capture. Our group have showed that FeOOH and TiO(OH)_2_ could be used as catalytic supports to reduce the activation energy of the NaHCO_3_ decomposition reaction and then enhance the decomposition rate^11,12^. Idem et al. reported that H-ZSM and γ-Al_2_O_3_ could be used to accelerate the amine based solvent regeneration and thus reducing the heat duty for amine regeneration^13,14^. Bhatti et al.^15^ recently reported an inspiring finding that transition metal oxides could affect spent monoethanolamine (MEA) regeneration. Two of those materials, MoO_3_ and V_2_O_5_, were found to improve desorption rates substantially because, in fact, they react and dissolve in the CO_2_-rich MEA solvent. For this reason, they cannot act as a classic, reusable catalyst. The other oxides investigated in that work, including TiO_2_, had only marginal effects.

In this work, we find a fundamentally different catalyst in structure—TiO(OH)_2_ that can radically change the pathway and thus lower the energy of CO_2_ capture. TiO(OH)_2_ turns out to be not only very effective in accelerating CO_2_ desorption—the key step in CO_2_ capture, but also quite stable, and thus a cost-effective CO_2_ capture catalyst. A proton transfer based catalytic CO_2_ capture mechanism is proposed. Also, the catalysis function of TiO(OH)_2_ is supported with both CO_2_ desorption experiments and characterization results.

## Results

### Catalytic effect of TiO(OH)_2_ on CO_2_ absorption and desorption

**Fig. 1:**
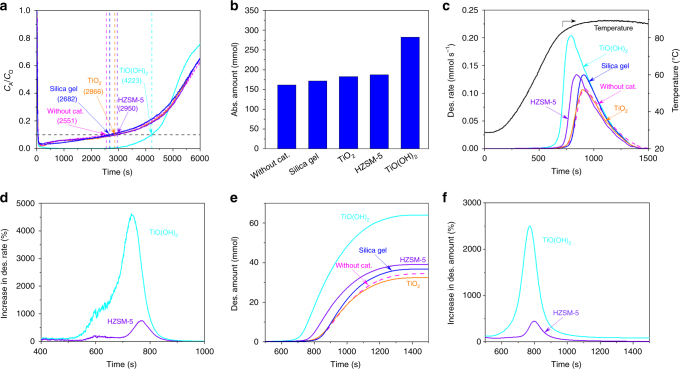
Effect of TiO(OH)_2_ catalyst on CO_2_ absorption (abs.) and desorption (des.). **a** Uncatalyzed and catalyzed CO_2_ absorption profiles of 20 wt% MEA solution. **b** The quantities of the CO_2_ sorbed within the effective absorption time (>90% CO_2_ capture). **c** The rates of CO_2_ desorption from spent 20 wt% MEA sorbent without and with uses of catalyst (cat.). **d** The percentage increases in CO_2_ desorption rate due to the use of TiO(OH)_2_. **e** Effects of TiO(OH)_2_ on the quantities of desorbed CO_2_. **f** The percentage increases in CO_2_ desorption amount due to the use of TiO(OH)_2_. Absorption conditions—total mass of solution: 200 g; MEA concentration in the solution: 20 wt%; total flow rate of gas: 1000 mL/min; composition of gas: 10 vol% CO_2_, 10 vol% O_2_, and 80 vol% N_2_; temperature: 25 °C; absorption time: 6000 s. Desorption conditions—Total mass of solution: 200 g; MEA concentration in the solution: 20 wt%; temperature: 88 °C; time: 2400 s

The catalytic effect of TiO(OH)_2_ on CO_2_ absorption, sorption for short, and desorption using MEA solution as an example is examined in this work with a setup shown in Supplementary Fig. 1 using synthetic flue gas mixture with 10 vol% CO_2_, 10 vol% O_2_, and 80 vol% N_2_. An optimal dosage (Supplementary Fig. 2) of 2 wt% TiO(OH)_2_ is used for all the tests. Figure 1a presents CO_2_ breakthrough curves for a 20 wt% MEA with and without TiO(OH)_2_, HZSM-5, silica gel, and TiO_2_, all at 2 wt%. Silica gel with similar particle size and porosity to TiO(OH)_2_ barely changes the CO_2_ sorption breakthrough profile. Qualitatively consistent with the Bhatti et al. work^15^, TiO_2_ also barely changes the CO_2_ sorption breakthrough curve. Also, recently reported H-ZSM5^13,14^ does not change CO_2_ breakthrough curve much. However, TiO(OH)_2_ significantly increases the effective sorption period in which capturing 90% CO_2_ in flue gas is realized as targeted by U.S. Department of Energy (DOE)^16^. The sorbent with longer effective period means that it has higher sorption efficiency and faster CO_2_ sorption. The effective sorption time without use of any catalyst and with the use of silica gel, TiO_2_, and HZSM-5 are only 2551, 2682, 2866, and 2950 s, respectively, ~12–16 % increase. While, the period due to use of TiO(OH)_2_ jumps to 4223 s, an increase by ~66% in comparison with that without catalyst. Accordingly, the quantities of the CO_2_ absorbed without and with uses of TiO(OH)_2_ within the corresponding effective sorption times are 162 and 283 mmol, respectively, as presented in Fig. 1b. This represents a 75% improvement.

The catalyst effect of TiO(OH)_2_ on CO_2_ desorption is evaluated with the spent MEA sorbent resulting from 6000 s of CO_2_ sorption (Fig. 1a). The CO_2_ desorption study is conducted by heating the CO_2_ spent MEA sorbent to a desired desorption temperature under the given heating profile shown in the right *y*-axis of Fig. 1c. The left *y*-axis of Fig. 1c shows the changes of the rates of CO_2_ desorption from spent 20 wt% MEA sorbent without and with uses of catalysts with time under the same temperatures. As shown in Fig. 1c, TiO_2_ barley changes CO_2_ desorption from the spent MEA sorbent. Unlike TiO_2_, the HZSM-5 can slightly improve CO_2_ desorption as reported by Idem et al.^13^. Silica gel also can slightly improve CO_2_ desorption, however it cannot lower the temperature for maximum CO_2_ desorption rate (Supplementary Table 1). Compared to the spent MEA without addition of the catalyst, the spent MEA with addition of TiO(OH)_2_ could desorb much more CO_2_ at the same low temperature range, and have a higher desorption rate at low temperatures. The highest desorption rate of spent MEA with the presence of TiO(OH)_2_ reaches 0.204 mmol s^−1^ at as early as 792 s (Supplementary Table 1), while the desorption rates of spent MEAs without the use of TiO(OH)_2_ and with the use of HZSM-5 are only 0.0162 mmol s^−1^ and 0.101 mmol s^−1^, respectively, at the same time point. The percentage increases in CO_2_ desorption rate due to the use of TiO(OH)_2_ and HZSM-5 catalysts as a function of time are presented in Fig. 1d. Remarkably, the increase in the rate of TiO(OH)_2_ catalyzed CO_2_ desorption can be as high as 4500%, while the highest increase in the rate of HZSM-5 catalyzed CO_2_ desorption is only 750%. Therefore, TiO(OH)_2_ can significantly catalyze CO_2_ desorption, lower CO_2_ desorption temperatures required for conventional CO_2_ capture technologies, make CO_2_ desorption occur at lower than 100 °C operable or practical, and significantly reduce the energy consumption of CO_2_ capture. The further extended practical implication of the high CO_2_ desorption rate could be its capability in reducing the dimension of amine scrubbing stripper for a given CO_2_ capture task.

Figure 1e shows the effects of TiO(OH)_2_ on the quantities of desorbed CO_2_ within the same-time-length cycle of CO_2_ sorption and desorption. The drastic enhancement in CO_2_ desorption kinetics is illustrated in Fig. 1e with an increase in the total quantity CO_2_ desorbed in the same time interval. Only 34.5 mmol CO_2_ is desorbed from the spent MEA solution when the catalyst was not used, and the increase of the amount of CO_2_ desorbed is ~0% when TiO_2_ is used. Silica gel shows a CO_2_ desorption amount of 36.9 mmol, a ~7.0% increase. The HZSM-5 slightly improves CO_2_ desorption amount to 39.2 mmol, only 14% increase. Extraordinarily, the presence of TiO(OH)_2_ leads to a desorption of 64.1 mmol CO_2_, about 86% increase. Although TiO(OH)_2_ and HZSM-5 have similar surface areas (Supplementary Table 3), the improvement in the quantity of CO_2_ desorption resulting from the use of TiO(OH)_2_ is 430% higher than that with HZSM-5. It should be mentioned that the difference in CO_2_ desorption amounts within a CO_2_ sorption-desorption cycle should be much larger than 86% if CO_2_ sorption is stopped when CO_2_ sorption efficiency falls below DOE-target, 90%. Percentage increase in CO_2_ desorption amount due to the use of TiO(OH)_2_ and HZSM-5 are provided in Fig. 1f. In comparison with the CO_2_ desorption of the uncatalyzed spent MEA, the cumulatively desorbed CO_2_ quantity of the catalyzed spent MEA with TiO(OH)_2_ is found to be 2500% higher than that of the uncatalyzed spent MEA at 771 s.

To avoid the impact of initial CO_2_ loading difference on CO_2_ desorption, the uncatalyzed and catalyzed CO_2_ desorption studies started with two MEA solvents containing the same amount of CO_2_ (~0.520 mol CO_2_ mol^−1^ MEA), which are achieved by absorbing CO_2_ for 6000 s without the use of TiO(OH)_2_. The desorption results without and with the use of TiO(OH)_2_ are summarized in Supplementary Fig. 3 and Table 1. Clearly, the presence of TiO(OH)_2_ catalyst is the main reason for the significantly accelerated CO_2_ desorption, given that the initial CO_2_ loadings in both spent MEA solvents are the same. The catalytic desorption profile of the spent MEA solvent shows a peak desorption rate of 0.183 mmol s^−1^, 168% of the 0.109 mmol s^−1^ achieved under uncatalyzed desorption condition. The increase in the CO_2_ desorption rate resulting from the use of TiO(OH)_2_ still can be as high as 2900% in comparison with that achieved without use of TiO(OH)_2_. Accordingly, the total amount of desorbed CO_2_ increases from 34.5 mmol to 53.5 mmol with the use of TiO(OH)_2_ (Supplementary Fig. 3C).

### Catalyst and sorbent stability

**Fig. 2:**
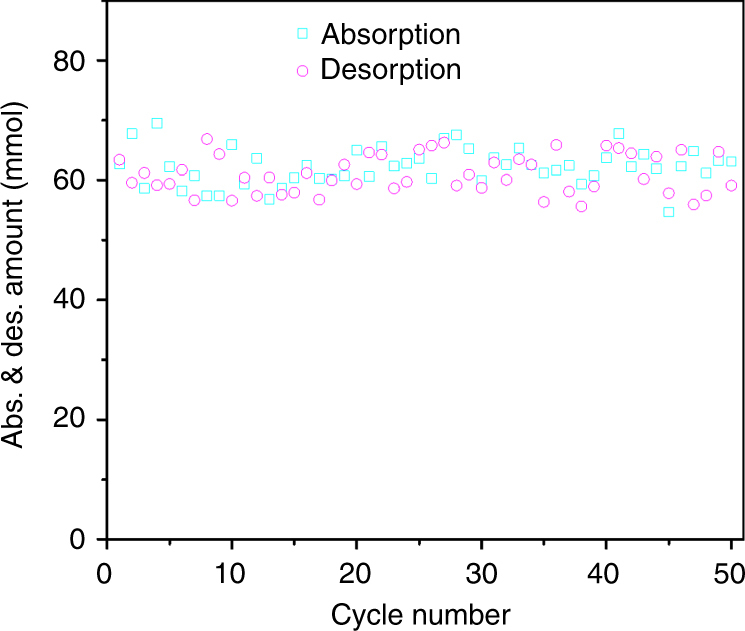
Catalyzed cyclic CO_2_ absorption and desorption. The cyclic tests indicate the stability of TiO(OH)_2_ under the given CO_2_ absorption and desorption conditions. Absorption conditions—total mass of solution: 200 g; MEA concentration in the solution: 20 wt%; total flow rate of gas: 1000 mL/min; composition of gas: 10 vol% CO_2_, 10 vol% O_2_, and 80 vol% N_2_; temperature: 25 °C; absorption time: 6000 and 3000 s for the 1st and 2nd–50th absorption tests, respectively. Desorption conditions—Total mass of solution: 200 g; MEA concentration in the solution: 20 wt%; temperature: 88 °C; time: 2400 s

The stability of TiO(OH)_2_ is assessed with 50 cycles of sorption-desorption tests. As shown in Fig. 2, no obvious decreases in both CO_2_ sorption and desorption with the cyclic tests are observed. After cyclic tests, the spent catalyst is filtered, washed, dried, and then characterized by X-ray diffraction (XRD), thermogravimetric analysis (TGA), scanning electron microscope (SEM), and transmission electron microscope (TEM) (Supplementary Figs. 4–6). These characterization results prove that chemical structure of the TiO(OH)_2_ is stable after 50 cyclic tests. The reason for the appearance of tiny XRD peaks of TiO_2_ with the spent TiO(OH)_2_ is not clear and need to be further studied. FT-IR result of the spent MEA solution (Supplementary Fig. 7) shows that MEA also remains stable during the cyclic tests, which may not be the case when it is exposed to the conventionally high regeneration temperatures.

### Raman spectroscopy

**Fig. 3:**
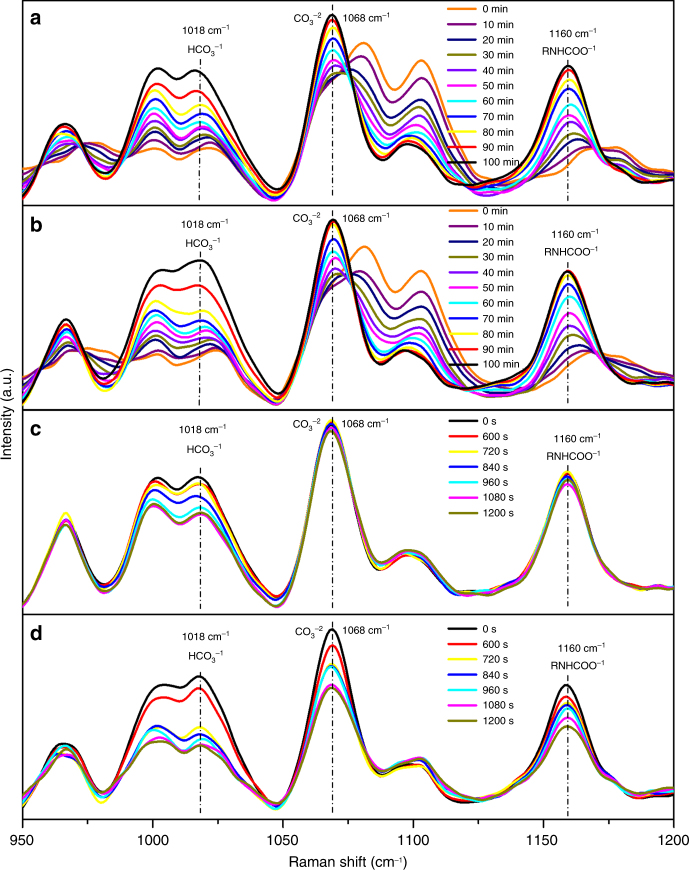
Raman spectra of solutions at different times. The indicated times are the periods when samples were taken during absorption and desorption tests for Raman spectrum analysis. The peak intensities are proportional to the concentration of species in solution. **a** CO_2_ absorption without TiO(OH)_2_, **b** CO_2_ absorption with TiO(OH)_2_, **c** CO_2_ desorption without TiO(OH)_2_, **d** CO_2_ desorption with TiO(OH)_2_. Absorption conditions—total mass of solution: 200 g; MEA concentration in the solution: 20 wt%; total flow rate of gas: 1000 mL/min; composition of gas: 10 vol% CO_2_, 10 vol% O_2_, and 80 vol% N_2_; temperature: 25 °C; absorption time: 6000 s. Desorption conditions–total mass of solution: 200 g; MEA concentration in the solution: 20 wt%; temperature: 88 °C; time: 2400 s

Raman spectroscopy is used to investigate the catalytic effect of TiO(OH)_2_ on both CO_2_ sorption and desorption. To observe the impact of the catalyst on the sorption step, CO_2_ sorption with and without TiO(OH)_2_ for different sorption periods is performed. The corresponding Raman spectra of the spent MEA sorbents are measured and presented in Fig. 3a and b. The bonds at 1018 cm^−1^, 1068 cm^−1^, and 1160 cm^−1^ are attributed to C–OH stretching of HCO_3_^−^, symmetric C–O stretching of CO_3_^2−^, and C–N stretching of RNHCO_2_^−^, respectively^17^. The peak height of RNHCO_2_^−^ generated under catalytic condition reached its highest point more quickly than that without use of the catalyst. Also, the peak intensity of HCO_3_^−^ at the end of the first 100 min of catalyzed CO_2_ sorption is stronger than that obtained without use of catalyst. Both Raman spectra observations clearly demonstrate the great effect of the catalyst on CO_2_ sorption. On the other hand, Raman spectra of the uncatalyzed and catalyzed CO_2_ desorption tests for different periods (0–1200 s) are conducted and the spectra of the resulting MEA solutions are measured and shown in Fig. 3c and d to evaluate the catalytic enhancement effect of TiO(OH)_2_. As can be seen from Fig. 3c, the peak intensity of HCO_3_^−^ in the uncatalyzed desorption derived MEA solution decreased gradually as CO_2_ desorption continues, and that of CO_3_^2−^ and RNHCO_2_^−^ does not change during the first 840 s and then started to decrease at a much slower rate with desorption time. However, when desorption is catalyzed, all peak heights of HCO_3_^−^, CO_3_^2−^, and RNHCO_2_^−^ in the spent MEA solutions decrease at higher rates. The peak intensity of HCO_3_^−^ in the TiO(OH)_2_-derived MEA solution becomes much weaker within just 720 s, a clear indication of a significant catalytic enhancement of CO_2_ desorption due to TiO(OH)_2_.

### Catalytic mechanism

**Fig. 4:**
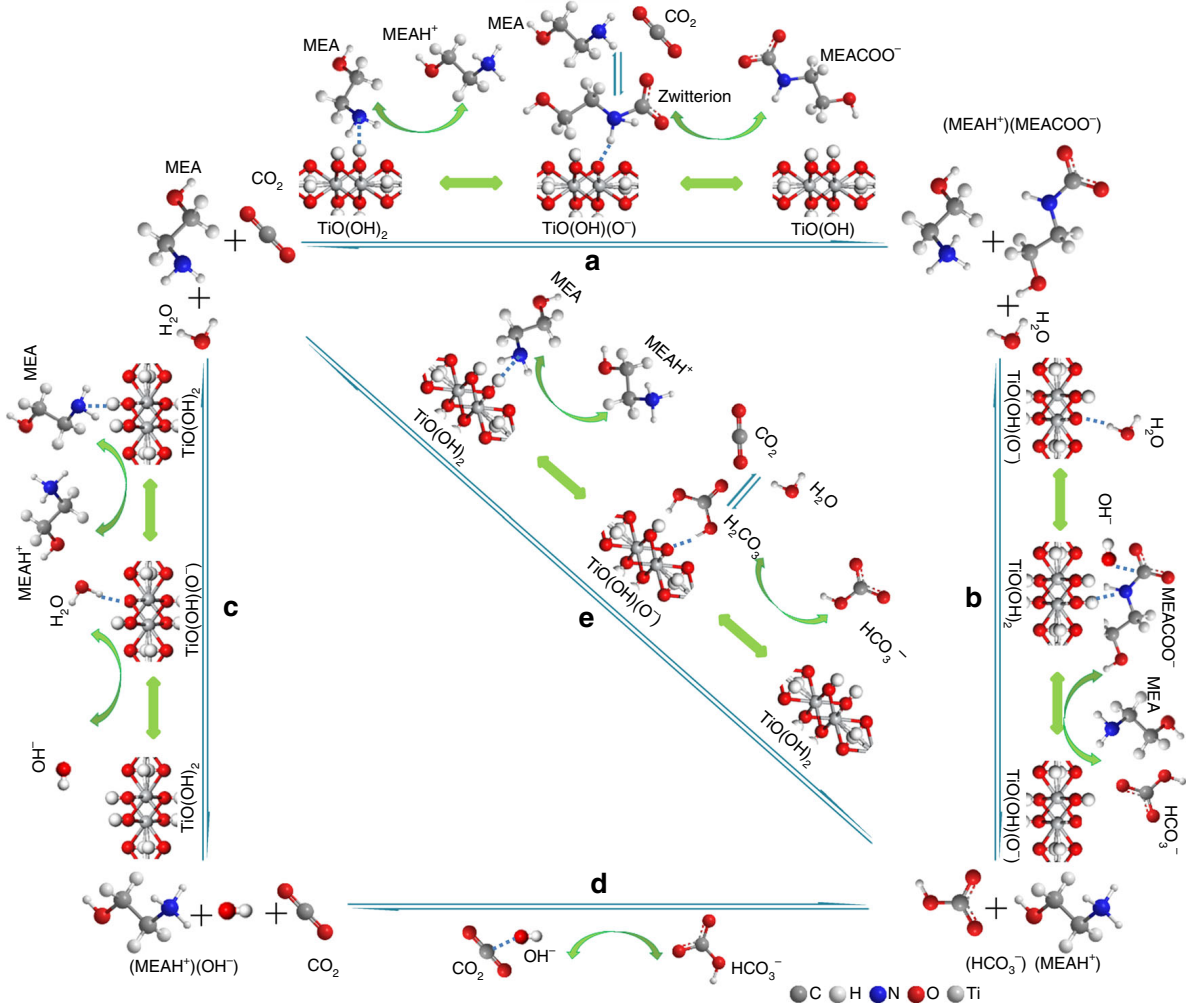
Proposed catalytic mechanism. Three possible pathways for TiO(OH)_2_ to accelerate CO_2_ absorption and desorption with MEA being a sorbent are presented. The hydroxyl group of TiO(OH)_2_ accelerates both protonation and deprotonation reactions and thus CO_2_ capture through reversible sorption and desorption via the special Lewis structure or the dual weak alkalinity and acidity of TiO(OH)_2_

A possible catalytic mechanism for TiO(OH)_2_-catalzyed MEA based CO_2_ sorption and desorption is proposed in Fig. 4. There are three possible pathways for bicarbonate formation during CO_2_ sorption and MEA regeneration (CO_2_ desorption) (Supplementary Fig. 8). In the first pathway, forward and reverse steps a and b include carbamate (MEACO_2_^−^) generation via a zwitterion intermediate, and then bicarbonate formation through hydrolysis^18^. In the second pathway, one proceeds with forward and reverse formation of (MEAH^+^)(OH^−^), and then bicarbonate, as shown in c and d steps for CO_2_ sorption and desorption, respectively. The last one essentially is the association and disassociation of carbonic acid (e and f steps). Protons transfers are directly associated with MEA based CO_2_ absorption and desorption^18^. The hydroxyl group of TiO(OH)_2_ with the ability of donating and accepting protons can greatly accelerate proton-involved reactions including protonation and deprotonation (Fig. 4). For example, TiO(OH)_2_ can catalyze steps a and b by providing protons for MEA and accepting protons from zwitterion, which is beneficial to the formation of MEAH^+^ and MEACO_2_^−^ during CO_2_ sorption, while it provides protons for MEACO_2_^−^ decomposition and accepts protons for MEAH^+^ deprotonation, which is favorable to sorbent regeneration or CO_2_ desorption. Special attention should be paid to critical role of TiO(OH)_2_ in the second and third pathways, in which the difficult direct proton transfer from MEAH^+^ to HCO_3_^−^ is avoided via its proton donation to HCO_3_^−^and deprotonation from MEAH^+^.

### Methyl diethanolamine based sorbent

To illustrate that the catalytic enhancement of TiO(OH)_2_ is not unique to MEA, we use another common amine solvent methyl diethanolamine (MDEA), as an example, as shown in Supplementary Fig. 9. Comparing Fig. 1 and Supplementary Fig. 9 suggests that TiO(OH)_2_ exhibits even stronger catalytic effect on CO_2_ desorption from MDEA. Only 2.12 mmol CO_2_ is desorbed from the spent 20 wt% MDEA solution when the catalyst is not used. By contrast, the presence of TiO(OH)_2_ leads to a desorption of 8.72 mmol CO_2_, about 311% increase.

## Discussion

In summary, TiO(OH)_2_ can significantly accelerate the both CO_2_ sorption and desorption, and lower temperature and thus energy requirements for CO_2_ capture. The alleviation in the capture conditions could lead to fundamental changes of people’s passion for CO_2_ emission control and successful fulfillment of Paris Agreement.

## Methods

### Preparation of TiO(OH)_2_

Titanium isopropoxide was used as the precursor for preparing TiO(OH)_2_ in this work. The first step was to add a predetermined amount of titanium isopropoxide into deionized (DI) water with a molar ratio of H_2_O:Ti(O-iC_3_H_7_)_4_ being 1400:1, followed by stirring the resulted mixture for 4 h. The precipitate powder was then filtered, rinsed three times with DI water and ethanol, then dried at 100 °C for ~10 h.

### Characterization

The nitrogen physisorption at 77 K was performed on a Quantachrome Quadrasorb SI to determine the surface areas and pore structure of TiO(OH)_2_. Powder X-ray diffraction (XRD) data were obtained by a Rigaku Smart Lab diffraction system with a Cu Kα radiation operated at 40 kV and 40 mA. Thermogravimetric analyses (TGA) of TiO(OH)_2_ were performed on a TA Instruments SDT Q600 with a heating ramp of 10 °C min^−1^. Fourier transform infrared (FTIR) spectroscopy spectra were collected on a Thermo Nicolet Magna-IR 760 spectrometer. Raman spectrum studies were conducted by using an Advantage 785 Raman Spectrometer with a 758 nm laser and up to 60 mW radiation power during measurement. The samples of MEA sorbents with different absorption and desorption times were prepared by taking about 1 ml solutions with a syringe at specific time, followed by filtering them with syringe filter to remove catalysts, and then placed them in clear shell vials for Raman measurements. Scanning electron microscopy (SEM) images of TiO(OH)_2_ were obtained by using a FEI Quanta FEG 450 field-emission scanning electron microscope. Transmission electron microscopy (TEM) images of TiO(OH)_2_ were taken by using a FEI Tecnai G2 F20 S-Twin 200 kV transmission electron microscope.

### CO_2_ capture

CO_2_ absorption and desorption studies were performed by using a setup schematically drawn in Supplementary Fig. 1. The reactor was a 500 mL glass reactor equipped with a magnetically coupled stirrer. In each sorption test, 200 g of 20 wt% MEA in water was used. Tests were done with or without catalyst. The concentrations (weight percentages) of catalysts varied from 1 wt% to 3 wt%.

CO_2_ absorption experiment was done at room temperature (~25 °C) and atmospheric pressure (0.78 bar at Laramie in Wyoming). A total of 20 wt% MEA solution was prepared by mixing ethanolamine with deionized water. Predetermined amounts of 20 wt% MEA and catalyst were added into the reactor with a stirring rate of 600 rpm. The simulated flue gas containing 10 vol% CO_2_, 10 vol% O_2_, and 80 vol% N_2_ was prepared by mixing individual gases. Three Parker mass flow controllers (Model 201) were used to accurately control the gas flow rates of CO_2_ (99.99%), O_2_ (99.999%), and N_2_ (99.999%) from the corresponding cylinders. The simulated flue gas with a total flow of 1000 ml min^−1^ was bubbled into MEA solution via a corrosion-resistant muffler (<100 μm, McMaster-Carr). The CO_2_ concentration of the outlet gas of the reactor was measured with an inline gas analyzer (NDIR ZRE, California Analytical Instruments), and the measured concentration-time profile was recorded by a data recording unit. The quantity of CO_2_ absorbed into MEA solution was calculated by integrating the recorded CO_2_ sorption profiles. Upon the completion of absorption step taking 6000 and 3000 s for fresh and cyclic MEA solutions, respectively, the valve for inlet gas was closed.

CO_2_ desorption was realized by heating the spent sorbent obtained from CO_2_ sorption step to a desired desorption temperature (88 °C) gradually. The desorbed CO_2_ went through a check valve and mixed with carrier gas (N_2_) with a flow rate of 500 mL min^−1^. The CO_2_ concentration of the gas mixture was measured by an in-line gas analyzer (NDIR ZRE, California Analytical Instruments). CO_2_ concentrations in the gas mixture and the corresponding temperatures of the spent MEA solution were recorded in the whole CO_2_ desorption process. Desorption test was stopped when the concentration of CO_2_ in gas mixture was lower than 0.01%. It should be noted the vaporized MEA during CO_2_ desorption operation was condensed and sent back to the reactor by using a condenser (#11 in Supplementary Fig. 1) along with a cooling unit (#13 in Supplementary Fig. 1). The following cyclic CO_2_ sorption started to proceed when the temperature of regenerated MEA solution was cooled to 25 °C.

CO_2_ absorption and desorption experiments with 20 wt% MDEA were conducted by using the same procedures as described earlier. In total 200 g of 20 wt% MDEA was used in the tests. After 6000 s of absorption, CO_2_ desorption was realized by heating the spent MDEA sorbent to a desired temperature with the heating profile used for CO_2_ desorption from spent MEA sorbent.

### Data availability

The data that support the findings of this study are available from the corresponding author upon request.

## Electronic supplementary material

Supplementary Information
