# Supplementary material for: Catalyst-TiO(OH)2 could drastically reduce the energy consumption of CO2 capture
Source: Nat Commun. 2018 Jul 10;9:2672. doi: 10.1038/s41467-018-05145-0 (PMC6039475; doi:10.1038/s41467-018-05145-0)
Supplement: Supplementary file 1 — Supplementary Information [file 41467_2018_5145_MOESM1_ESM.pdf]

Supplementary Information for

Catalyst-$TiO(OH)_2$ could drastically reduce the energy consumption of CO_2 capture

Qinghua Lai, Sam Toan, Mohammed A. Assiri, Huaigang Cheng, Armistead G Russell, Hertanto Adidharma, Maciej Radosz, Maohong Fan*

*Corresponding author. Email: mfan@uwyo.edu and mfan3@mail.gatech.edu.

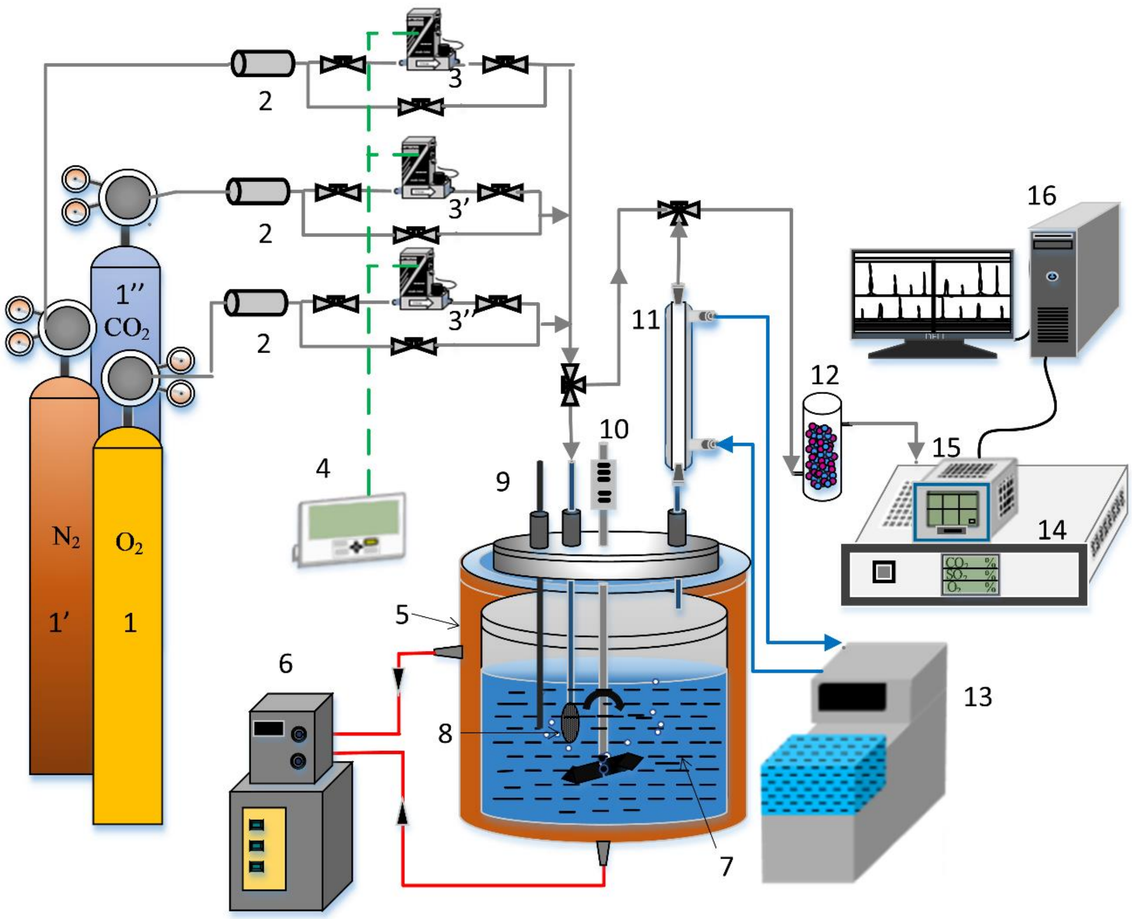

Supplementary Figure 1. Schematic drawing of MEA CO_2 absorption/desorption experimental setup. 1, 1, 1”: gas cylinders; 2: filter; 3: mass flow controller; 4: mass flow controller control module; 5: furnace; 6: thermostatic water bath; 7: catalyst suspended in the solvent; 8: muffler for inlet gas; 9: thermocouple; 10: mechanical stirrer; 11: condenser; 12: moisture remover; 13: cooling unit; 14: gas analyzer; 15: data recorder; 16: computer.

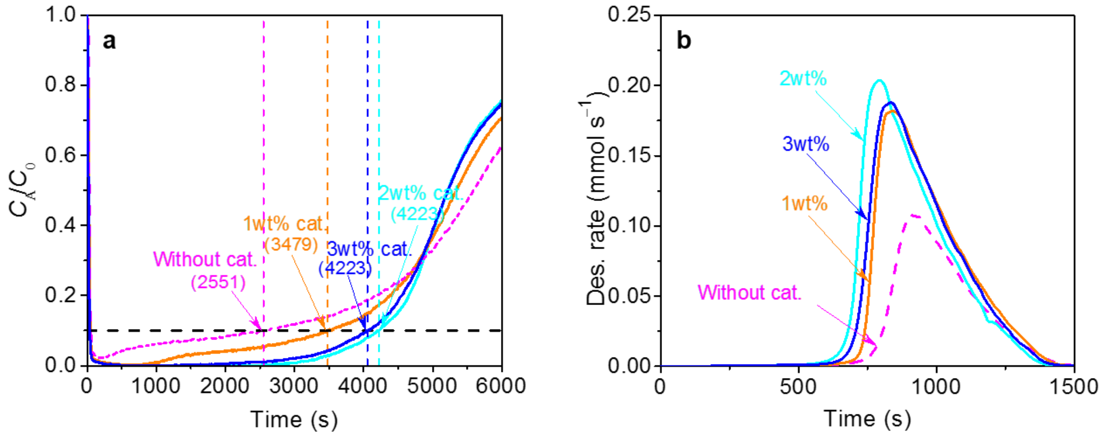

Supplementary Figure 2. Effect of $TiO(OH)_2$ catalyst (cat.) concentration on uncatalyzed and catalyzed CO_2 absorption (abs.) and desorption (des.). **a** CO_2 absorption profiles of 20 wt% MEA sorbent. **b** CO_2 desorption rate of spent 20 wt% MEA sorbent.

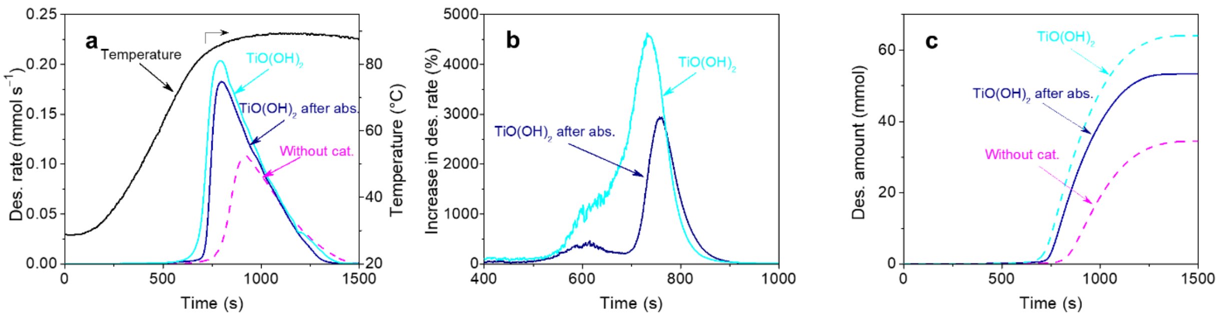

Supplementary Figure 3. Effect of addition of $TiO(OH)_2$ before and after absorption step on catalyzed CO_2 desorption. **a** The rates of CO_2 desorption from spent 20 wt% MEA sorbent without and with addition of $TiO(OH)_2$ before and after absorption step. **b** The percentage increases in CO_2 desorption rate due to the addition of $TiO(OH)_2$ before and after absorption step. **c** Effects of addition of $TiO(OH)_2$ before and after absorption step on the quantities of desorbed CO_2.

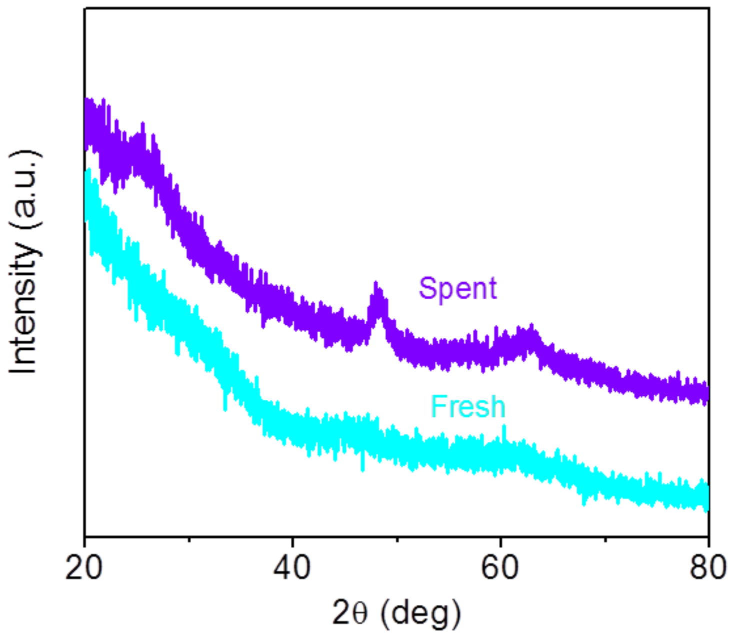

Supplementary Figure4. XRD results of the fresh and used $TiO(OH)_2$.

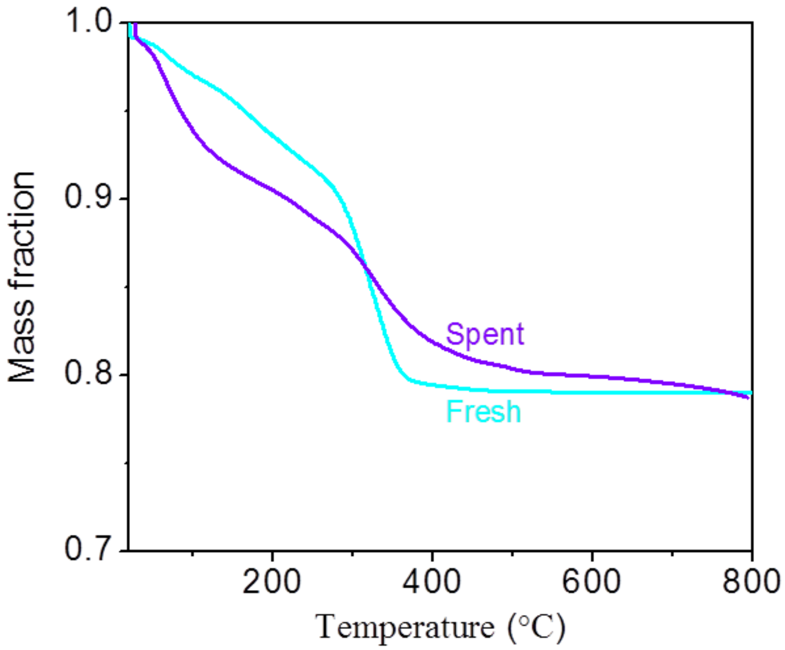

Supplementary Figure 5. TGA profiles of fresh and used $TiO(OH)_2$.

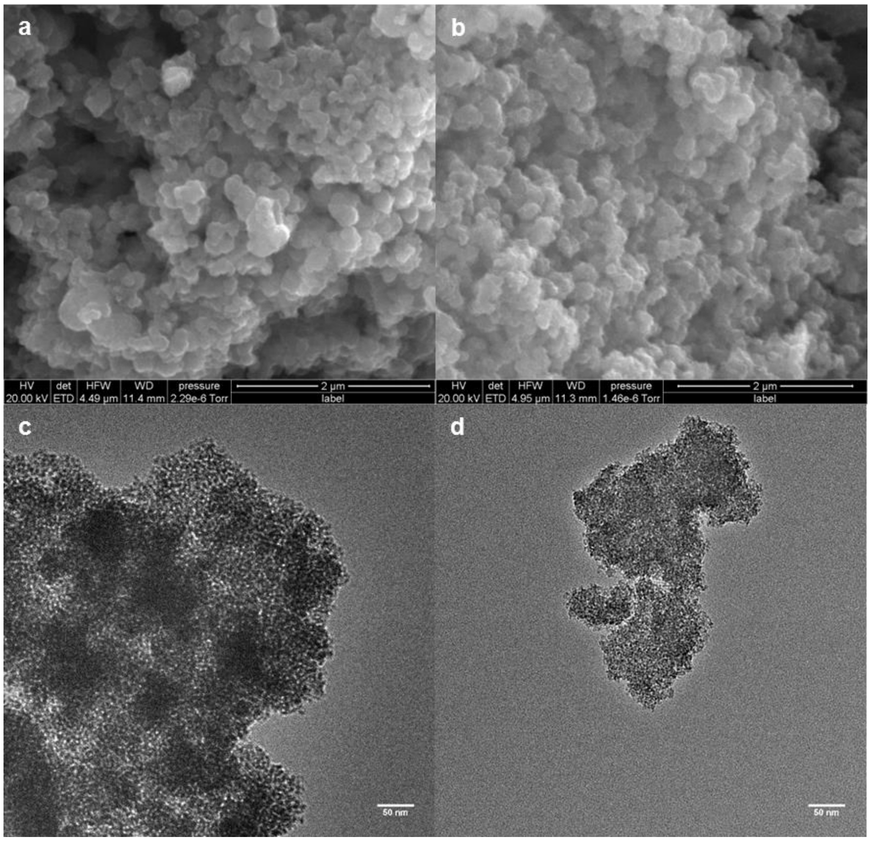

Supplementary Figure 6. Morphologies of fresh and used $TiO(OH)_2$. **a** SEM images of fresh and **b** used $TiO(OH)_2$. **c** TEM images of fresh and **d** used $TiO(OH)_2$.

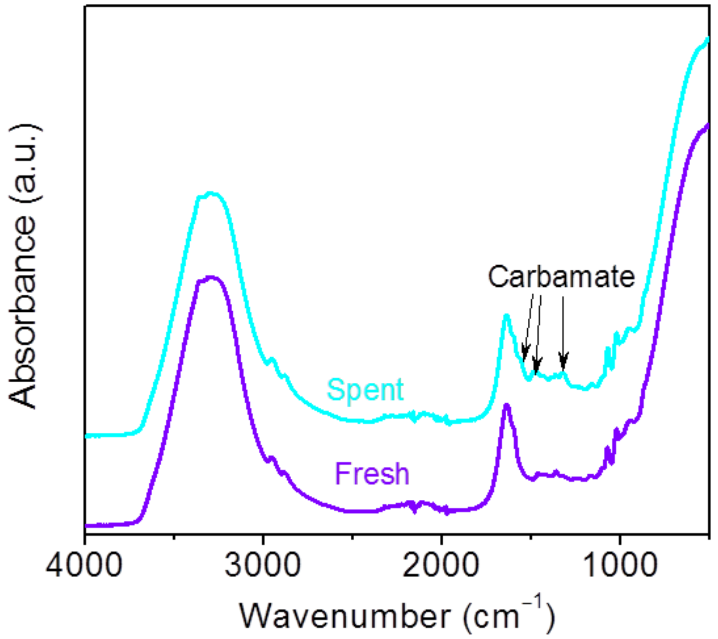

Supplementary Figure 7. FT-IR spectra of fresh and spent MEA sorbents.

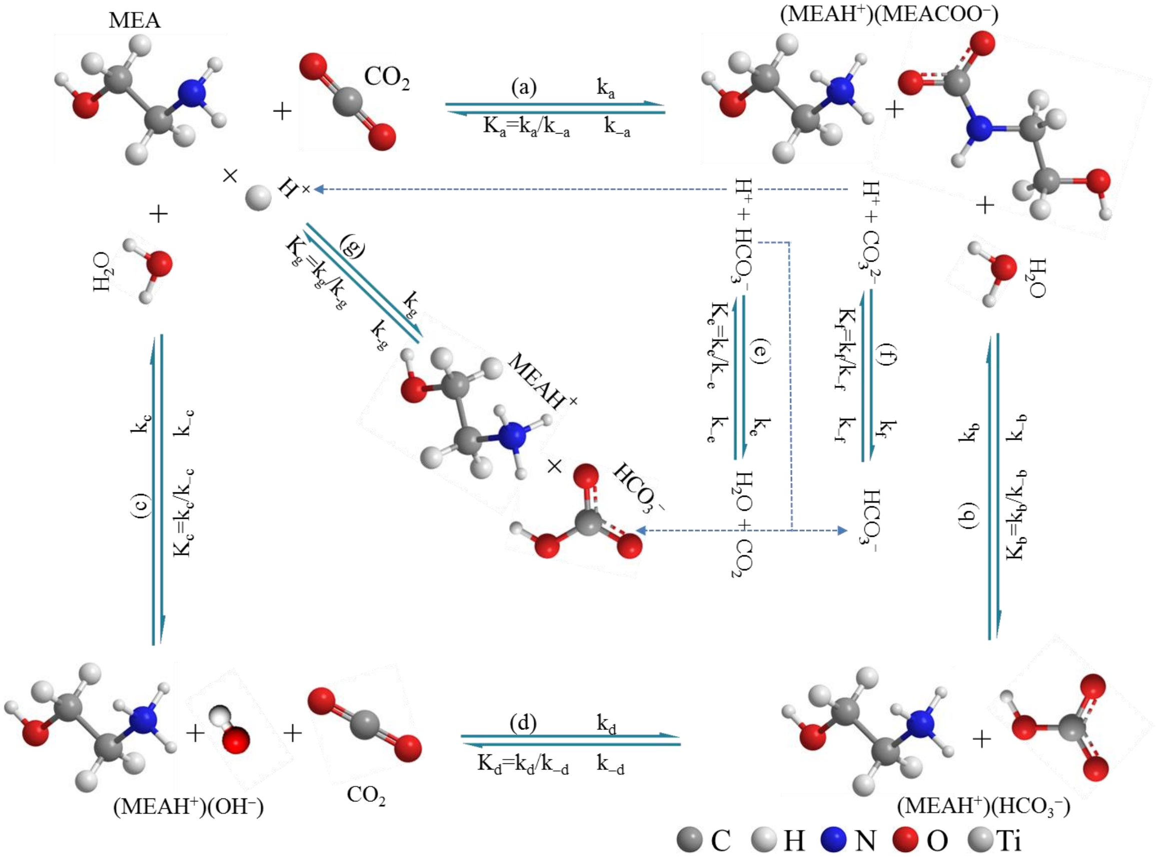

Supplementary Figure 8. Reaction pathways for uncatalyzed CO_2 absorption and desorption with MEA solution.

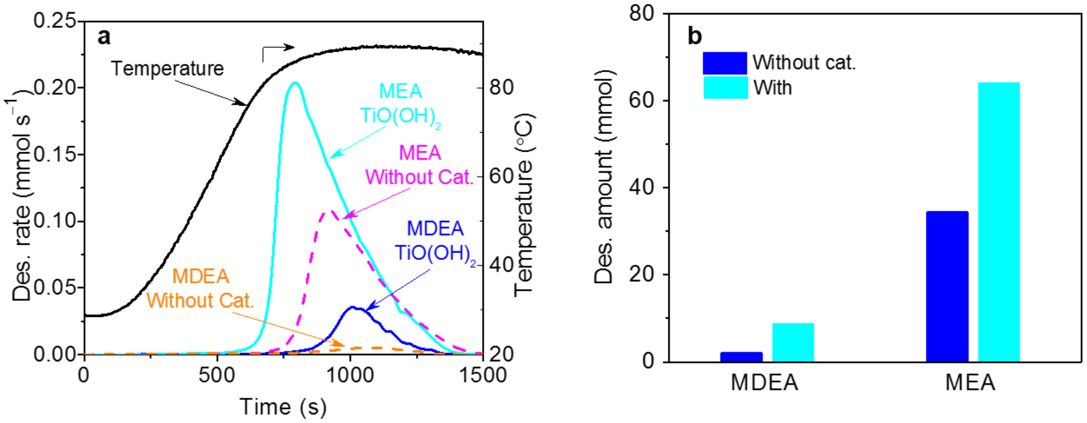

Supplementary Figure 9. CO_2 desorption amount of different 20 wt% amine-based sorbents with and without $TiO(OH)_2$. **a** CO_2 desorption rate. **b** CO_2 desorption amount.

Supplementary Table 1. Desorption rate, time, and temperature at maximum CO_2 desorption point.

MEA solvents	Maximum CO_2 des. rate (mmol s^{-1})	Time at maximum CO_2 des. rate (s)	Temperature at maximum CO_2 des. rate (°C)
Without Cat.	0.109	940	88.2
With TiO_2	0.107	898	88.0
With silica gel	0.133	910	88.2
With HZSM-5	0.133	839	87.0
With $TiO(OH)_2$	0.204	792	85.8
Add $TiO(OH)_2$ after abs.	0.183	797	85.8

Supplementary Table 2. CO_2 loading before and after desorption step.

MEA solvents	CO_2 loading after ads. step (mol CO_2 mol^{-1} MEA)	CO_2 loading after des. step (mol CO_2 mol^{-1} MEA)	sorbent cyclic capacity (mol CO_2 mol^{-1} MEA)
Without Cat.	0.520	0.467	0.0527
With TiO_2	0.519	0.469	0.0497
With silica gel	0.518	0.462	0.0563
With HZSM-5	0.522	0.462	0.0599
With $TiO(OH)_2$	0.541	0.443	0.0979
Add $TiO(OH)_2$ after abs.	0.520	0.438	0.0817

Supplementary Table 3. BET analysis results of fresh and used $TiO(OH)_2$.

Samples	Specific surface area (m^2 g^{-1})	Pore volume (cm^3 g^{-1})	Pore diameter (Å)
Fresh $TiO(OH)_2$	783.2	0.573	17.1
HZSM-5	580.6	0.122	19.1
TiO_2	48.44	0.084	15.3
Silica gel	532.8	0.760	24.4
